# Physicochemical Properties, Antioxidant and Cytotoxic Activities of Crude Extracts and Fractions from *Phyllanthus amarus*

**DOI:** 10.3390/medicines4020042

**Published:** 2017-06-18

**Authors:** Van Tang Nguyen, Jennette A. Sakoff, Christopher J. Scarlett

**Affiliations:** 1 School of Environmental and Life Sciences, Faculty of Science and Information Technology, University of Newcastle, Ourimbah, NSW 2258, Australia; C.Scarlett@newcastle.edu.au; 2 Department of Food Technology, Faculty of Food Technology, Nha Trang University, No. 2 Nguyen Dinh Chieu, Nha Trang 8458, Vietnam; 3 Department of Medical Oncology, Calvary Mater Newcastle Hospital, Waratah, NSW 2298, Australia; Jennette.Sakoff@newcastle.edu.au

**Keywords:** *Phyllanthus amarus*, extracts, fractions, physicochemical, antioxidant, cytotoxicity

## Abstract

**Background:** *Phyllanthus amarus* (*P. amarus*) has been used as a medicinal plant for the prevention and treatment of chronic ailments such as diabetes, hepatitis, and cancer. **Methods:** The physicochemical properties, antioxidant and cytotoxic activities of crude extracts and fractions from *P. amarus* were determined using spectrophotometric method. **Results:** The *P. amarus* methanol (*PA*M) extract had lower levels of residual moisture (7.40%) and water activity (0.24) and higher contents of saponins, phenolics, flavonoids, and proanthocyanidins (1657.86 mg escin equivalents, 250.45 mg gallic acid equivalents, 274.73 mg rutin equivalents and 61.22 mg catechin equivalents per g dried extract, respectively) than those of the *P. amarus* water (*PA*W) extract. The antioxidant activity of *PA*M extract was significantly higher (*p <* 0.05) than that of the *PA*W extract, *PA*M fractions, and phyllanthin (known as a major compound in the *P. amarus*). Higher cytotoxic activity of *PA*M extract based on MTT assay on different cell lines including MiaPaCa-2 (pancreas), HT29 (colon), A2780 (ovarian), H460 (lung), A431 (skin), Du145 (prostate), BE2-C (neuroblastoma), MCF-7 (breast), MCF-10A (normal breast), and U87, SJ-G2, SMA (glioblastoma) was observed in comparison to the *PA*W extract and *PA*M fractions. The cytotoxic potential of the *PA*W extract (200 μg/mL), based on the CCK-8 assay on a pancreatic cancer cell line (MiaCaPa2) was significantly lower (*p* < 0.05) than those of gemcitabine (50 nM) and a saponin-enriched extract from quillajia bark at 200 μg/mL (a commercial product), but was significantly higher than that of phyllanthin at 2 μg/mL. **Conclusions:** The results achieved from this study reveal that the *PA* extracts are a potential source for the development of natural antioxidant products and/or novel anticancer drugs.

## 1. Introduction

Cancer is a leading cause of death worldwide, accounting for 8.2 million deaths in 2012, in which pancreatic cancer (PC) is the fourth leading cause of cancer deaths in Western societies, with an overall 5-year survival of less than 7%, and the figures have not changed for several decades [1,2,3,4]. Although much research in PC treatment has been performed in recent years, PC is still a therapeutic challenge. Currently, the pancreatectomy is the only cure for PC in the early stages. Other treatment methods such as chemo, targeted, and radiotherapies are also utilized in later stages of PC. In regards to chemotherapy, current chemotherapeutic agents such as gemcitabine (a purine analog), fluoropyrimidine, and/or platinum drugs have been used. Further, erlotinib is an epidermal growth factor receptor tyrosine kinase inhibitor, which is used as a supplement to gemcitabine [1]. Pyrrolidine dithiocarbamate or disulfiram synergistically inhibited PC cell proliferation when used in combination with gemcitabine and enhancement by zinc ions [5]. A four-drug combination, named FOLFIRINOX (oxaliplatin, irinotecan, flurouracil, and leucovorin) demonstrated superiority in PC treatment with a median overall survival of 11 months, while median overall survival of 2, 6, 6–7, and 8.5 months for PC treatment with no therapy, gemcitabine alone, gemcitabine plus capecitabine or erlotinib, and gemcitabine plus abraxane or nab-paclitaxel, respectively [2,6]. However, the major challenge of these anticancer drugs is that they are highly toxic with limited treatment results, and are associated with degradation in the quality of life.

Over the past 30 years, about 45% of all anticancer drugs have been derived directly or indirectly from plant compounds, of which 12% are natural products and 32% are semi-synthetic derivatives of natural related products, with a number of plant-sourced agents in currently clinical use for the treatment of various cancers, such as taxanes from the *Taxus brevifolia* tree, vinca alkaloids from the *Catharanthus roseus* leaves, camptothecin analogs from the *Camptotheca acuminata* tree, and podophyllotoxin analogs from the *Podophyllum peltatum* plants [7]. Some plant-derived anticancer agents have been used for the treatment of pancreatic cancer, such as irinotecan, docetaxel, paclitaxel, triptolide, and minnelide (semi-synthetic) [8,9]. The traditional use of plants as folk medicine for cancer prevention and treatment in China, Ayurveda, Greco-Arabic traditions, European, Anthrosophical medicine, and North American traditions is also reported [10]. Therefore, the use of herbal medicines for cancer prevention and treatment is of great interest to researchers and patients because of lower toxicity and high availability [3].

*Phyllanthus amarus* (*P. amarus*) is broadly distributed in tropical and subtropical areas and used as a traditional herbal plant. Previous studies showed that the *P. amarus* extract contains a wide range of bioactive compounds, such as polyphenols, flavonoids, proanthocyanidins, triterpenes, saponins, lignans (phyllanthin), ellagitannins, sterols, and alkaloids [11,12], which display potent pharmacological activities, particularly anti-hepatitis [13,14], antioxidant [15,16,17], and anticancer activities [18,19,20,21].

Up to now, the physicochemical and biological properties of *P. amarus* (*PA*) extracts and fractions have not been sufficiently reported. For example, the antioxidant capacity of *PA* was determined for dried samples and extracts using assay of free radical scavenging capacity [17,21], but not for fractions from *PA* extract, while cytotoxic activity of *PA* extracts has been evaluated on several human cancer cell lines (colon: Caco-2 [18], lung: A549 and breast: MCF-7 [19,20], and hepatoma: HepG2 [21]) using 3-(4,5-dimethylthiazol-2-yl)-2,5-diphenyltetrazolium bromide (MTT) assay, but not focused on pancreatic cancer cell lines. With this in mind, our current study aimed to determine the physicochemical properties as well as to evaluate the antioxidant and cytotoxic activities of crude extracts and fractions from *PA* extract. Antioxidant analysis involved assays of free radical scavenging capacity and ferric reducing antioxidant power, while cytotoxic evaluation involved MTT assays and a Dojindo cell counting kit-8 (CCK-8), both targeting pancreatic cancer cell lines, to evaluate the potential application of *PA* extracts in the nutraceutical, medical, pharmaceutical, and cosmetic industries.

## 2. Materials and Methods

### 2.1. Plant Material

The whole plant of *Phyllanthus amarus* was collected in January 2015 from Hon Nghe, Vinh Ngoc, Nha Trang city, Khanh Hoa province, Vietnam and authenticated by Quang Ngai Union of the Science and Technology Association, Vietnam. Briefly, the fresh samples were placed in sealed plastic bags, covered with ice to minimize oxidation, and rapidly taken to the laboratories at the Nha Trang University. The fresh samples were then rinsed in deionized water to remove sand, soil, and foreign materials, drained, and dried by infrared exposure at 30 °C to constant weight in an infrared drying cabinet (SHN-L, Nha Trang University, Vietnam [15]) packaged in the vacuum sealed polyamide bags, and stored at −20 °C until required. Residual moisture of dried *P. amarus* sample was analyzed based on the Association of Official Analytical Chemists (AOAC) official methods of analysis [22] in a hot-air oven (Anax Pty Ltd., Sydney, NSW, Australia) at 120 °C to constant weight.

### 2.2. Analytical Chemicals

In this study, all chemicals were of analytical grade. Folin–Ciocalteu reagent, 2,2′-Azino-bis(3-ethylbenzothiazoline-6-sulphonic acid) (ABTS), 2,2-diphenyl-1-picryl-hydrazil (DPPH), gallic acid, rutin, catechin, ostruthin, gemcitabine, trolox, neocuproine, 2,4,6-tripyridyl-*s*-triazine (TPTZ), saponin-enriched quillaja bark extract, and iron (III) chloride were products of Sigma-Aldrich Pty Ltd. (Castle Hill, Sydney, NSW, Australia). Sulfuric acid and hydrochloric acid were purchased from Ajax Finechemicals (Sydney, NSW, Australia). Acetic acid was obtained from BDH Laboratory Supplies (Poole, England). Vanillin, potassium persulfate, sodium nitrite, methanol, and acetonitrile were purchased from Merck (Darmstadt, Germany). Sodium acetate trihydrate was obtained from Government Stores Department (Sydney, NSW, Australia). Ammonium acetate was purchased from BDH Chemicals (Boronia, VIC, Australia). Copper (II) chloride was obtained from Standard Laboratories (Maribyrnong, VIC, Australia). Aluminium chloride was purchased from Acros (Morris Plains, NJ, USA). Sodium carbonate anhydrous was obtained from Chem-supply (Adelaide, South Australia, Australia). Sodium hydroxide was purchased from Ajax chemicals (Sydney, NSW, Australia). 3-(4,5-dimethylthiazol-2-yl)-2,5-diphenyltetrazolium bromide (MTT), Dulbecco’s Modified Eagle’s Medium (DMEM) and Keratinocyte Serum-Free Medium (KSFM) were products of Gibco by Life Technologies (Grand Island, NY, USA). Dojindo Cell Counting Kit-8 (CCK-8) was obtained from Dojindo Molecular Technologies, Inc. (Rockville, MD, USA).

### 2.3. Preparation of Powdered P. amarus Extracts

*PA* extracts were prepared according to the previously described methods [17,23]. Briefly, 10 g of dried sample was soaked in 1000 mL of deionized water (for water extract) or 100% methanol (for methanol extract) at room temperature (21 ± 1 °C) for 20 min, followed by microwave-assisted extraction in a microwave oven (Sharp Carousel, Sharp Corporation, Bangkok, Thailand) at a power of 600 W, an irradiation time of 4 s/min and an extraction time of 50 min for water extract, or a power of 360 W, an irradiation time of 7 s/2 min and an extraction time of 40 min for methanol extract. After that, the extracts were rapidly cooled to room temperature in an ice water bath and filtered through qualitative No. 1 filter papers (Bacto Laboratories Pty Ltd., Mount Pritchard, NSW, Australia). The extracts were then evaporated under reduced pressure (−10 mbar) at 40 °C using a rotavapor (Buchi, Flawil, Switzerland) and dried to constant weight in a freeze drier (Rietschle Thomas Australia Pty Ltd., Seven Hills, NSW, Australia) at −45 °C, 0.1 atm for 48 h to obtain the powdered *PA* extracts, and stored at -20 °C until required. For the antioxidant capacity determination of the *PA* extracts, the powdered *PA* extracts were redissolved in 100% methanol at a concentration of 200 μg/mL using a fine vortex mixer (FINEPCR, Gyeonggi-do, Korea) until the solutions became completely clear and no little particles in suspension was observed.

### 2.4. Determination of Physicochemical Properties of Powdered P. amarus Extracts

#### 2.4.1. Residual Moisture and Water Activity

The residual moisture of the *PA* extracts was analyzed based on the AOAC official methods of analysis [22] at 100 °C to constant weight in a hot-air oven (Anax Pty Ltd., Sydney, NSW, Australia). Water activity (a_w_) of *PA* extracts was measured at room temperature using a water activity meter (Pa_w_kit, Decagon Devices, Washington, DC, USA).

#### 2.4.2. Total Phenolic Content (TPC)

The TPC of the *PA* extracts was quantified based on the previous study [24] with some modifications. To 0.5 mL of the extract was mixed with 2.5 mL of 10% (*v*/*v*) Folin–Ciocalteu reagent in distilled water. The mixture was kept for 6 min, then 2 mL of 7.5% (*w*/*v*) Na_2_CO_3_ solution was added. The absorbance of the mixture was measured at 765 nm using a UV-VIS spectrophotometer (Cary 50 Bio, Mulgrave, VIC, Australia) after incubation in the dark at room temperature for 1 h. Methanol was used as a control. TPC was quantified as mg gallic acid equivalents (GAE)/g dried extract.

#### 2.4.3. Total Flavonoid Content (TFC)

The TFC of the *PA* extracts was analyzed using the formerly reported method [24]. 0.5 mL of the extract was mixed with 2 mL of distilled water and 0.15 mL of 5% (*w*/*v*) NaNO_2_ solution. The mixture was then kept in the dark at room temperature for 6 min. After that, 0.15 mL of 10% (*w*/*v*) AlCl_3_ solution was added and the mixture was kept for 6 min. Finally, 2 mL of 4% (*w*/*v*) NaOH solution and 0.7 mL of distilled water were added and the mixture was incubated for 15 min. The absorbance of mixture was measured at 510 nm. Methanol and rutin were used as a control and standard, respectively. TFC was calculated as mg of rutin equivalents (RE)/g dried extract.

#### 2.4.4. Proanthocyanidin Content

The proanthocyanidin content of the *PA* extracts was estimated based on the previously presented method [24]. To 0.5 mL of the extract was mixed with 3 mL of 4% (*w*/*v*) vanillin solution and 1.5 mL of concentrated HCl. The mixture was kept in the dark at room temperature for 15 min before measuring absorbance at 500 nm. Methanol and catechin were used as a control and standard, respectively. Proanthocyanidin content was estimated as mg of catechin equivalents (CE)/g dried extract.

#### 2.4.5. Saponin Content (SC)

The saponin content (include several sapogenins like sterols and steroids) of the *PA* extracts was determined according to the prior work [17]. To 0.5 mL of the extract was mixed with 0.5 mL of 8% (*w*/*v*) vanillin solution and 5 mL of 72% (*v*/*v*) H_2_SO_4_ solution. The mixture was kept at 70 °C for 15 min and then rapidly cooled by an ice water bath to room temperature. The absorbance of the mixture was measured at 560 nm. Methanol was used as a control. Saponin content was quantified as mg of escin equivalents (EE)/g dried extract.

#### 2.4.6. Isolation of Major Fractions from *P. amarus* Methanol (*PA*M) Extract

Based on the results of physicochemical analysis above, we selected the *PA*M extract for the separation of major fractions for further evaluation of their antioxidant and cytotoxic activities. A Shimadzu HPLC system (Shimadzu, Chiyoda-ku, Tokyo, Japan was used for isolation of major fractions from the *PA*M extract based on the previously established method [25]. Basically, the original *PA*M extract after filtering through qualitative filter paper and evaporating under reduce pressure was filtered through 0.45 μm nylon membranes (Phenex syringe filters) and then 300 μL of the extract was injected by an auto sampler (SIL-10AV, Shimadzu, Kyoto, Japan) onto a Semi-prep Phenomenex Synergy 4U Polar-RP 80A column (250 mm × 10 mm) (Phenomenex, Torrance, CA, USA). The column was kept at 35 °C in an oven thermal sphere (Phenomenex, Torrance, CA, USA) and coupled to an auto fraction collector (FRC-10A, Shimadzu, Kyoto, Japan). The mobile phase included two solvents: 0.2% orthophosphoric acid in distilled water (A) and 100% acetonitrile (B). The flow rate was 3 mL/min with the gradient as follows: 0–5 min, 0% B; 5–20 min, 20% B; 20–30 min, 30% B; 30–48 min, 30% B; 48–53 min, 0% B. Bioactive compounds were detected at 210 nm using a diode array detector (8PD-M20A, Shimadzu, Kyoto, Japan). Isolation of major fractions from the *PA*M extract was achieved based on retention time of these compounds. Nine major fractions were separately collected from the fraction collector (284 runs in total). These fractions were then evaporated under reduced pressure (−30–50 mbar) at 45–60 °C using a rotavapor (Buchi, Flawil, Switzerland), dried to a constant weight in a freeze drier (Rietschle Thomas Australia Pty Ltd., Seven Hills, NSW, Australia) at −45 °C, 0.1 atm for 72 h to obtain the powdered *PA*M factions, and stored them at −20 °C until used for further assessment.

### 2.5. Determination of Antioxidant Capacity of Powdered P. amarus Extracts and Fractions

#### 2.5.1. ABTS Radical Scavenging Capacity (ARSC)

The ARSC of the *PA* extracts and fractions was evaluated using the formerly established method [26] with some modifications. Briefly, a stock solution was prepared by mixing 7.4 mM ABTS^•+^ and 2.6 mM K_2_S_2_O_8_ solutions (1:1 ratio), kept in the dark at room temperature for 12 h, and stored at −20 °C. Prior to use, mixing 1 mL of stock solution with 60 mL of methanol was performed to obtain a working solution with absorbance of 1.1 ± 0.02 at 734 nm. 0.15 mL of the extract was mixed with 2.85 mL of the working solution and incubated in the dark at room temperature for 2 h. The absorbance of mixture was measured at 734 nm. Methanol and ostruthin were used as a control and positive control, respectively. The results were calculated as mg trolox equivalents (TE)/g dried sample.

#### 2.5.2. DPPH Radical Scavenging Capacity (DRSC)

The DRSC of the *PA* extracts and fractions was measured based on the previous study [24] with some modifications. Briefly, 0.024% (*w*/*v*) DPPH in methanol was prepared to make a stock solution and stored at −20 °C. Before use, 1.0 mL of stock solution was diluted with 45 mL of methanol to obtain a working solution with absorbance of 1.1 ± 0.02 at 515 nm. 0.15 mL of the extract was mixed with 2.850 mL of the working solution and incubated in the dark at room temperature for 3 h. The absorbance of mixture was measured at 515 nm. Methanol and ostruthin were used as a control and positive control, respectively. The results were estimated as mg trolox equivalents (TE)/g dried sample.

#### 2.5.3. Ferric Reducing Antioxidant Power (FRAP)

The FRAP of the *PA* extracts and fractions was estimated based on the former report [27] with some modifications. Three regents were prepared including 300 mM acetate buffer solution at pH 3.6 (reagent A); 10 mM TPTZ solution in 40 mM HCl (reagent B); and 20 mM FeCl_3_·6H_2_O solution (reagent C). Prior to use, reagents A, B, and C were mixed at a ratio of 10:1:1 to make a fresh FRAP solution. 0.15 mL of the extract was mixed with 2.850 mL of the fresh FRAP solution and incubated in the dark at room temperature for 30 min. The absorbance of mixture was read at 593 nm. Methanol and ostruthin were used as a control and positive control, respectively. The results were calculated as mg trolox equivalents (TE)/g dried sample.

### 2.6. Assessment of Cytotoxic Potential of Powdered P. amarus Extracts and Fractions

#### 2.6.1. Growth Inhibition of Cancer Cell Lines by *PA* Extracts

The cytotoxic potential of *PA* extracts was assessed according to a previously reported methods [4,28] based on the growth inhibition of *PA* extracts on eleven cancer cell lines including MiaPaCa-2 (pancreas), HT29 (colon), A2780 (ovarian), H460 (lung), A431 (skin), Du145 (prostate), BE2-C (neuroblastoma), MCF-7 (breast), and U87, SJ-G2, SMA (glioblastoma) and one non-cancer derived cell line MCF-10A (normal breast). Briefly, all cancer cell lines were cultured in DMEM supplemented with 10% fetal bovine serum, 50 IU/mL penicillin, 50 μg/mL streptomycin, and 2 mM l-glutamine. The MCF-10A cells were cultured in DMEM:F12 (1:1) cell culture media, 5% heat inactivated horse serum, supplemented with 50 IU/mL penicillin, 50 μg/mL streptomycin, 20 mM Hepes, 2 mM L-glutamine, 20 ng/mL epidermal growth factor, 500 ng/mL hydrocortisone, 100 ng/mL cholera toxin, and 10 μg/mL insulin. Cells were plated in triplicate in 100 μL DMEM on a 96-well plate, at a density of 2500–4000 cells per well. When cells were at logarithmic growth after 24 h, medium without (control) and with the *PA* extracts (100 μL) was added to each well to give a final concentration of 100 μg/mL (day 0). MTT assay was employed where absorbance was read at 540 nm to determine growth inhibition after 72 h of incubation based on the difference between the optical density values on day 0 and those at the end of drug exposure. Cell growth inhibition as a percentage was determined where a value of 100% is indicative of total growth inhibition, while a value greater than 100% is indicative of growth inhibition and cell death.

#### 2.6.2. Viability of Pancreatic Cancer and Normal Cells by Treatment with *P. amarus* Methanol (*PA*M) Extract and Fractions

##### Cell Culture

A human pancreatic cancer cell line (MiaPaCa_2_) and a normal human pancreatic ductal epithelial (HPDE) cell line were cultured based on the previously described methods [3,4] with some modifications. Briefly, the cells were grown in DMEM supplemented with 10% fetal bovine serum (FBS), 2.5% horse serum, and 1% L-glutamine for MiaPaCa_2_ or KSFM supplemented with 2.5 μg epidermal growth factor (EGF) human recombinant and 25 mg bovine pituitary extract (BPE) for HPDE at 37 °C with 5% CO_2_, respectively.

##### Cell Viability

Cell viability was determined using the CCK-8 assay based on the formerly reported methods [3,4] with some modifications. Briefly, cells were seeded into a 96 well plate at 3 × 10^3^ cells per well for MiaPaCa_2_ or 7 × 10^3^ cells per well for HPDE and allowed to adhere for 24 h at 37 °C with 5% CO_2_. Media was removed and the cells were then treated with various concentrations of the *PA*M extract in the media (200–12.5 μg/mL), its fractions (50 μg/mL), ostruthin (20 μg/mL), and gemcitabine (50 nM) as positive controls, quillaja bark extract (200 μg/mL) as a comparative sample, and 0.5% DMSO as a control. After 72 h incubation at 37 °C with 5% CO_2_, the media was removed and 100 μL of 10% CCK-8 solution in the media was added and incubated at 37 °C with 5% CO_2_ for 2 h. Absorbance was measured at 450 nm and cell viability was expressed as a percentage of control.

### 2.7. Statistical Analysis

All experiments were carried out at least in triplicate. The results were analyzed using SPSS software (Version 22, SPSS Inc., Chicago, IL, USA) and expressed as means ± standard deviations. Statistical comparisons were made using one-way analysis of variance (ANOVA) and Tukey HSD post hoc tests. *p*-values at least 0.05 were considered to be significantly different.

## 3. Results and Discussion

### 3.1. Physicochemical Properties of Powdered P. amarus Extracts

Residual moisture and water activity of powdered extracts affect greatly their stability and storability. Table 1 shows that residual moisture and water activity of the powdered *PA* extracts ranged from 7.40 to 8.85% and 0.24 to 0.35, respectively, revealing that the freeze drying method used in this study was a suitable method for preparing the powdered *PA* extracts. The residual moisture of the powdered *PA* extracts obtained in this study was lower than that of saponin-enriched bitter melon powder (9.2–12.1%), while its water activity was comparable to that of bitter melon powder (0.2–0.4) [29]. Chirife and Fontana [30] reported that water activity in the range from 0.45 to 0.68 can inhibit the proliferation of microorganisms and enzyme activity, leading to increase storability of samples. Table 1 also shows that the *PA*M extract was richer in saponins, phenolics, flavonoids, and proanthocyanidins (1657.86 mg escin equivalents, 250.45 mg gallic acid equivalents, 274.73 mg rutin equivalents, and 61.22 mg catechin equivalents/g dried extract, respectively) than those of the *PA*W extract (1037.34 mg escin equivalents, 86.89 mg gallic acid equivalents, 158.17 mg rutin equivalents, and 37.05 mg catechin equivalents/g dried extract, respectively), which were much higher than those of bitter melon powder (77.1–113.6 mg escin equivalents, 17.0–22.4 mg gallic acid equivalents, 2.8–5.8 mg rutin equivalents, and 1.9–3.2 mg catechin equivalents/g dried powder, respectively) [29]. Previous studies reported that phenolics display potent antioxidant activity [16,31], while saponins possess great anticancer capacity [32,33]. The results from this study have given a hypothesis that the PA extracts may be a potential source against oxidation and cancer, which are assessed and presented in the next sections.

### 3.2. Major Fractions Isolated from P. amarus Methanol (PAM) Extract

**Figure 1:**
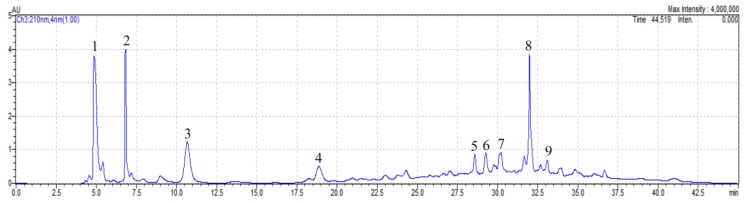
HPLC chromatogram of *P. amarus* methanol (*PA*M) extract with PDA detector set at 210 nm.

Figure 1 shows the HPLC chromatogram of the *PA*M extract detected at 210 nm. Based on the retention time of bioactive compounds, nine major fractions, numbered F1 to F9, were isolated from the *PA*M extract and collected using an auto fraction collector. These included: F1: 4.72–5.40 min, F2: 6.65–7.11 min, F3: 10.10–11.20 min, F4: 18.40–19.35 min, F5: 28.40–28.85 min, F6: 29.02–29.60 min, F7: 30.00–30.50 min, F8: 31.75–32.34 min, and F9: 32.90–33.30 min. The dried weight of the powdered *PA*M fractions after freeze drying were obtained as follows: F1: 1.093 g, F2: 0.925 g, F3: 1.507 g, F4: 1.401 g, F5: 0.858 g, F6: 0.801 g, F7: 0.695 g, F8: 1.059 g, and F9: 0.534 g, which were then used for the assessment of antioxidant and cytotoxic activities. However, the *PA*M fractions have not yet been identified in this study, therefore further study may be required to characterize these *PA*M fractions and purify them for further applications. Nguyen at al. [4] also used a HPLC system coupled to an auto fraction collector to isolate completely four major fractions from *P. trimera* root methanol extract for assessing their antioxidant and cytotoxic capacities.

### 3.3. Antioxidant Capacity of Powdered P. amarus Extracts and Fractions

**Figure 2:**
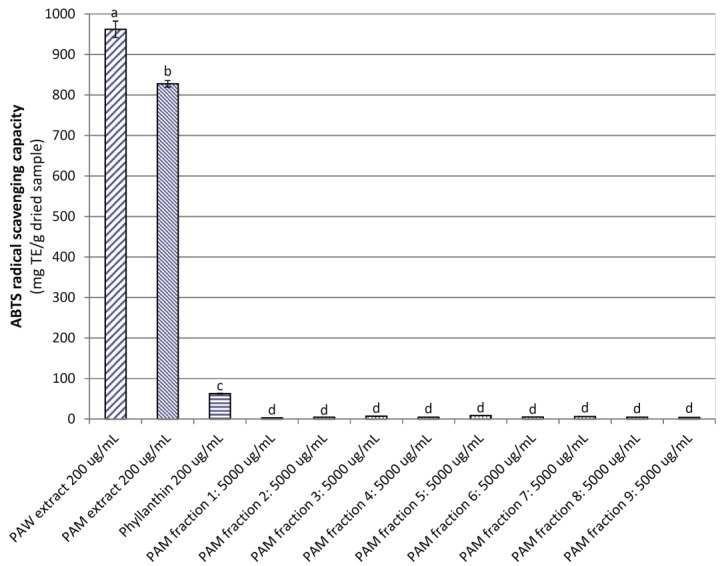
ABTS radical scavenging capacity of powdered *P. amarus* water (*PA*W) and methanol (*PA*M) extracts and *PA*M fractions. Phyllanthin was used as a positive control. Different letters within the columns denotes a significant difference between treatments (*p <* 0.05).

Figure 2 shows ABTS radical scavenging capacity (ARSC) of *PA* extracts, nine fractions from the *PA*M extract and phyllanthin. At 200 μg/mL, the ARSC of the *PA*W extract (962.20 mg TE/g dried sample) was significantly higher (*p <* 0.05) than those of the *PA*M extract, phyllanthin, and all nine *PA*M fractions (827.74, 62.89, and 2.62–8.75 mg TE/g dried sample, respectively), indicating that phyllanthin and the *PA*M fractions displayed weak antioxidant capacity when compared to *PA* extracts. Potent ARSC of both *PA* extracts was likely contributed by high contents of phenolics, flavonoids, and proanthocyanidins (86.89 mg GAE, 158.17 mg RE and 37.05 mg CE/g dried extract for *PA*W extract; and 250.45 mg GAE, 274.73 mg RE and 61.22 mg CE/g dried extract for *PA*M extract, respectively) [16].

**Figure 3:**
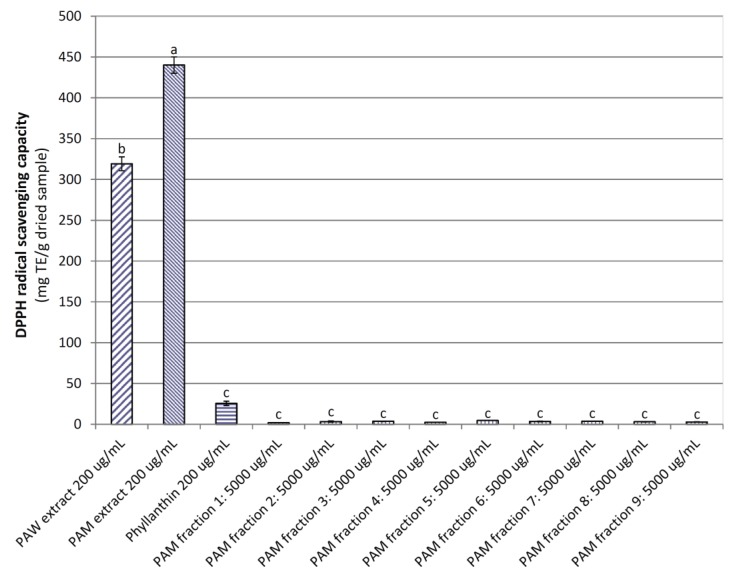
The DPPH radical scavenging capacity of powdered *P. amarus* water (*PA*W) and methanol (*PA*M) extracts and *PA*M fractions. Phyllanthin was used as a positive control. Different letters within the columns denotes a significant difference between treatments (*p <* 0.05).

In contrast, the DPPH radical scavenging capacity (DRSC) of the *PA*M extract (440.13 mg TE/g dried sample) was significantly higher (*p <* 0.05) than those of the *PA*W extract, phyllanthin, and the nine *PA*M fractions (319.14, 25.62, and 1.89–4.68 mg TE/g dried sample, respectively; Figure 3). The higher contents of phenolics, flavonoids, and proanthocyanidins in the *PA*M extract mainly contributed to its stronger DRSC when compared to the *PA*W extract [34]. Previous studies have also indicated a positive correlation between phenolics, flavonoids, and proanthocyanidins and the DRSC of *P. trimera* root [35] and *P. amarus* [16]. The study [36] showed the *P. amarus* extract and phyllanthin concentrations for maximum DRSC were 300 μg/mL and 20 μmol/mL, with the IC_50_ being 162.3 μg/mL versus 7.4 μmol/mL.

**Figure 4:**
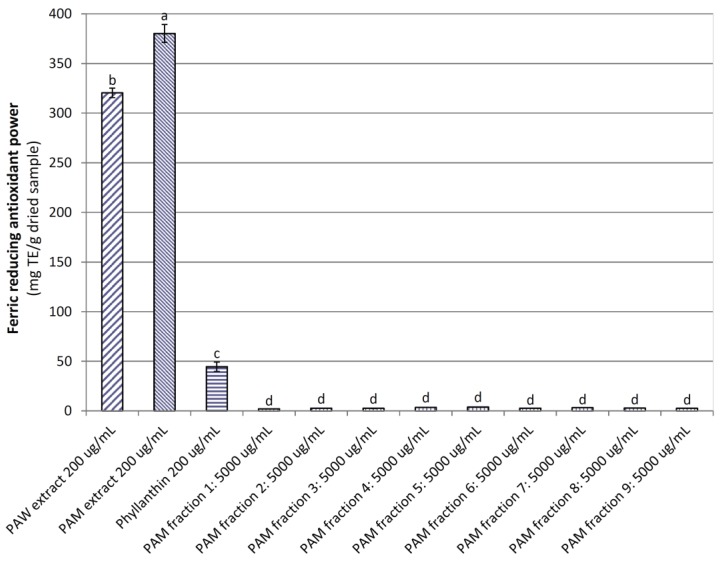
Ferric reducing antioxidant power of powdered the *P. amarus* water (*PA*W) and methanol (*PA*M) extracts and *PA*M fractions. Phyllanthin was used as a positive control. Different letters within the columns denotes a significant difference between treatments (*p <* 0.05).

Similar to the DRSC, the ferric reducing antioxidant power (FRAP) of the *PA*W extract, phyllanthin, and nine *PA*M fractions (320.39, 44.57, and 2.14–3.83 mg TE/g dried sample) was significantly lower (*p <* 0.05) than that of the *PA*M extract (380.22 mg TE/g dried sample; Figure 4). Strong FRAP of both *PA* extracts would have also been as a consequence of their high phenolic, flavonoid, and proanthocyanidin contents. This outcome is supported by previous studies, which reported that the FRAP of the extracts had a positive correlation with their TPC, for example, *E. robusta* leaf [28], *E*. *tirucalli* [37], and *P. amarus* [16].

The findings from this study indicated that the *PA* extracts displayed potent antioxidant activity against free radicals and for reducing ferric ion, while all fractions from the *PA*M extract exhibited weak antioxidant capacity. This may be due to a loss of activity of the *PA*M fractions during the fractionation, evaporation, and drying processes by the oxidation and decomposition of sensitive bioactive compounds under the effect of UV light, oxygen in the environment, and/or the deterioration of key bioactive compounds by concentrated orthophosphoric acid remaining in the fractions. Therefore, the *PA* extracts are considered as a promising source for the development of potential antioxidant products.

### 3.4. Cytotoxic Potential of Powdered P. amarus Extracts and Fractions

#### 3.4.1. Growth Inhibition of Cancer Cell Lines by *PA* Extracts

Table 2 shows the cell growth inhibition of the *PA* extracts on twelve cell lines, including MiaPaCa-2 (pancreas), HT29 (colon), A2780 (ovarian), H460 (lung), A431 (skin), Du145 (prostate), BE2-C (neuroblastoma), MCF-7 (breast), MCF-10A (normal breast), and U87, SJ-G2, SMA (glioblastoma). At a dose of 100 μg/mL, cell growth inhibition of the *PA*M extract on all cell lines was much higher than that of the *PA*W extract, likely due to the *PA*M extract possessing much higher contents of saponins, phenolics, flavonoids, and proanthocyanidins when compared to the *PA*W extract.

Cell growth inhibition of the *PA*M extract achieved in this study was comparable to those of the extracts from *E. robusta* leaf [28] and maroon bush (*S. spinescens*) [38], but it was weaker than those from *P. trimera* root [4] and leaf [7]. Lee at el. [19] evaluated the anti-metastatic potential of *Phyllanthus* spp. (*P. niruri*, *P. urinaria*, *P. watsonii*, and *P. amarus*) on lung (A549) and breast (MCF-7) carcinoma cells and found that *Phyllanthus* extracts effectively reduced invasion, migration, and adhesion for both MCF-7 and A549 cells at concentrations ranging from 20–200 and 50–500 μg/mL for methanolic and aqueous extracts, respectively, while Abhyankar et al. [20] determined the cytotoxic effects of the methanolic extract from *P. amarus* hairy roots on the human breast adenocarcinoma cell line (MCF-7) and a cervical cancer cell line (HeLa), and indicated that the cytotoxicity correlated well with the increased levels of intracellular reactive oxygen species (ROS) and decreased mitochondrial membrane potential, particularly an appreciable anti-proliferative effect of the *P. amarus* hairy root extract on the MCF-7 cells was recorded via induction of apoptosis. Lawson-Evi et al. [18] also reported that the aqueous extract of *P. amarus* was more cytotoxic on human colon cancer cells (Caco-2) than a hydroalcoholic extract, with IC_50_ values being 89.6 μg/mL and 277 μg/mL for the *P. amarus* aqueous and hydroalcoholic extracts, respectively.

The outcomes obtained from this study allow further application of the *PA*M extract alone or in combination with other agents for the development of novel anti-cancer drugs.

#### 3.4.2. Viability of Pancreatic Cancer and Normal Cells by treatment with *P. amarus* Methanol (*PA*M) Extract and Its Fractions

**Figure 5:**
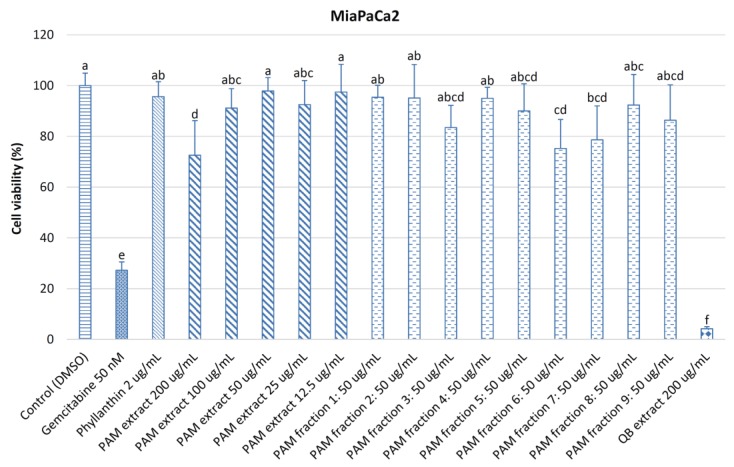
Viability of MiaPaCa2 cells treated with different concentrations of powdered *P. amarus* methanol (*PA*M) extract and fractions. DMSO was used as a control; gemcitabine and phyllanthin were used as positive controls; and quillajia bark (QB) extract was used as a comparative sample. Different letters within the columns denotes a significant difference between treatments (*p <* 0.05).

Figure 5 shows the cell viability of pancreatic cancer cells MiaPaCa2 treated with various concentrations of the *PA*M extract (200–12.5 μg/mL), nine fractions from the *PA*M extract (50 μg/mL), phyllanthin (2 μg/mL), gemcitabine (50 nM), and quillaja bark extract (200 μg/mL). At 200 μg/mL of the *PA*M extract, cell viability of MiaPaCa2 cells (72.58%) was significantly lower (*p <* 0.05) than those at lower concentrations of 100–12.5 μg/mL (91.15–97.90%), 50 μg/mL of nine *PA*M fractions (75.24–95.42%), and 2 μg/mL of phyllanthin (95.67%), but it was significantly higher (*p <* 0.05) than those of 50 nM of gemcitabine (27.22%) and quillaja bark extract at 200 μg/mL (4.20%). This indicated that a component in fraction 6 might have a great contribution to the cytotoxicity on MiaPaCa2 cells of the *PA*M extract. Therefore, fraction 6 should be purified and characterized in further studies. Cell viability of MiaPaCa2 cells treated with 200 μg/mL of the *PA*M extract in this study was much higher than those treated with 200 μg/mL of lilly pilly (*S. paniculatum*) extract [39] (about 23%), 200 μg/mL or water and methanol *E. tirucalli* extracts (approximately 30 and 7.0%, respectively) [37] and 200 μg/mL of methanol extracts from *P. trimera* root [4] and leaf [7] (4.2 and 6.6%, respectively).

**Figure 6:**
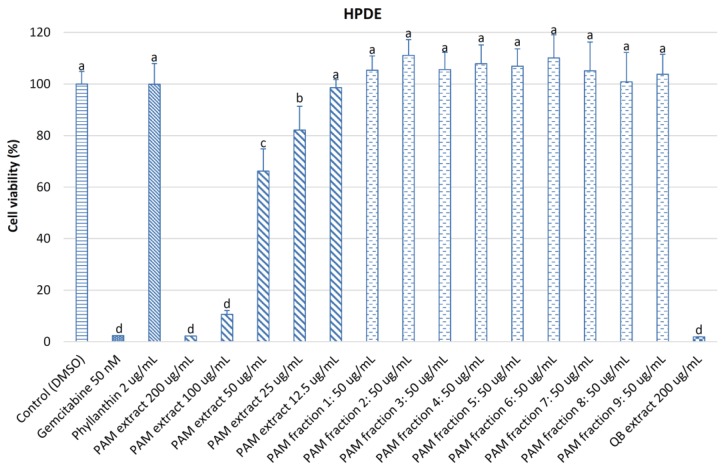
Viability of HPDE cells treated with different concentrations of powdered *P. amarus* methanol (*PA*M) extract and fractions. DMSO was used as a control; gemcitabine and phyllanthin were used as positive controls; and quillajia bark (QB) extract was used as a comparative sample. Different letters within the columns denotes a significant difference between treatments (*p <* 0.05).

Figure 6 indicates that the cell viability of normal human pancreatic ductal epithelial (HPDE) cells treated with 200 and 100 μg/mL of the *PA*M extract (2.18 and 10.61%, respectively), 50 nM of gemcitabine (2.37%), and 200 μg/mL of quillaja bark extract (1.88%) was significantly lower (*p <* 0.05) than those treated with the *PA*M extract at 50–12.5 μg/mL (66.20–98.57%), nine fractions from the *PA*M extract (100.85–111.14%), and phyllanthin (99.91%), revealing that the nine fractions isolated from the *PA*M extract (except fraction 6) exhibited weak activity on MiaPaCa2 and HPDE cells. The reason for this may be due to a loss of activity of the *PA*M fractions during the fractionation, evaporation, and drying processes by the oxidation and decomposition of sensitive bioactive compounds under the effect of UV light and oxygen in the environment and/or the deterioration of key bioactive compounds by concentrated orthophosphoric acid remaining in the fractions. Low cytotoxicity on HPDE cells of the *PA*M fractions is a prominent advantage for further applications. The cell viability of HPDE cells treated with 100 μg/mL of the *PA*M extract found in this study (10.61%) was much lower than that treated with 100 μg/mL of *S. paniculatum* extract [39] (58%) but it was much higher than those treated with 100 μg/mL of methanol extracts from *P. trimera* root [4] and leaf [7] (2.4 and 2.2%, respectively). The *PA*M extract greatly affected cell growth of MiaPaCa2 and HPDE cells as it was rich in saponins, phenolics, flavonoids, and proanthocyanidins.

This is the first report describing the effect of the *PA*M extract against pancreatic cancer cells, therefore further study should be continued to evaluate the anti-pancreatic cancer potential of the *PA*M extract on other pancreatic cancer cell lines, as well as to understand the mechanism of action of the *PA*M extract on these pancreatic cancer cell lines.

## 4. Conclusions

This study showed the great antioxidant potential of the *PA* extracts in vitro, in which the *PA*M extract processed higher antioxidant and cytotoxic capacities as compared to the *PA*W extract. In addition, the antioxidant and cytotoxic activities of the crude *PA* extracts were stronger than those of the fractions. Therefore, we can conclude that the *PA* extracts are a promising source for further applications in the nutraceutical, medical, and pharmaceutical industries for the development of natural antioxidant products.

## Figures and Tables

**Table 1:** Physicochemical properties of powdered *P. amarus* water and methanol extracts.

Properties	Water Extract	Methanol Extract
Residual moisture (%)	8.85 ± 0.74 *	7.40 ± 0.14
Water activity (aw)	0.35 ± 0.02	0.24 ± 0.02
Total phenolic content (mg GAE/g dried extract)	86.89 ± 6.06	250.45 ± 11.93
Total flavonoid content (mg RE/g dried extract)	158.17 ± 14.38	274.73 ± 1.56
Proanthocyanidin content (mg CE/g dried extract)	37.05 ± 6.03	61.22 ± 13.94
Saponin content (mg EE/g dried extract)	1037.34 ± 52.17	1657.86 ± 441.62

* Means and standard deviations were of triplicate. GAE: Gallic acid equivalents; RE: Rutin equivalents; CE: Catechin equivalents; EE: Escin equivalents.

**Table 2:** Cytotoxic capacity of powdered *P. amarus* (*PA*) extracts on different cancer cell lines

Cancer Cell Line (Cell Type)	% Cell Growth Inhibition at 100 μg/mL of *PA* Extracts (*n* = 3–6)	
	Water (*PA*W) Extract	Methanol (*PA*M) Extract
MiaPaCa2 (pancreas)	38 ± 5	48 ± 3
HT29 (colon)	23 ± 3	49 ± 7
A2780 (ovarian)	46 ± 4	74 ± 3
H460 (lung)	16 ± 5	53 ± 3
A431 (skin)	31 ± 1	74 ± 2
Du145 (prostate)	26 ± 8	73 ± 3
BE2-C (neuroblastoma)	51 ± 14	54 ± 4
MCF-7 (breast)	16 ± 4	67 ± 8
MCF-10A (breast-normal)	43 ± 7	73 ± 2
U87 (glioblastoma)	23 ± 6	62 ± 10
SJ-G2 (glioblastoma)	28 ± 1	54 ± 10
SMA (glioblastoma-murine)	49 ± 18	31 ± 13
